# Preclinical Evaluation of Fenbendazole for Controlling *Gyrodactylus kobayashii* (Monogenea, Gyrodactylidae) in Goldfish: Dose Optimization and Safety Assessment

**DOI:** 10.3390/ani15121811

**Published:** 2025-06-19

**Authors:** Jing Dong, Jiangtao Li, Yongtao Liu, Qiuhong Yang, Ning Xu, Xiaohui Ai, Shun Zhou

**Affiliations:** 1 Yangtze River Fisheries Research Institute, Chinese Academy of Fishery Sciences, Wuhan 430223, China; dongjing@yfi.ac.cn (J.D.);; 2 Freshwater Fisheries Research Center, Chinese Academy of Fishery Sciences, Wuxi 214081, China

**Keywords:** preclinical study, fenbendazole, monogenean, anthelmintic treatment, aquaculture

## Abstract

This preclinical study investigated the efficacy and safety of fenbendazole, a broad-spectrum benzimidazole anthelmintic, for the treatment of *Gyrodactylus kobayashii* in goldfish (*Carassius auratus*). In vivo bath treatments demonstrated potent, dose-dependent anthelmintic efficacy, achieving 98.58% efficacy at a concentration of 0.02 mg/L and a 48 h EC_50_ of 0.006 mg/L. A short-duration (6 h) bath at 0.06 mg/L, followed by an 18 h recovery period in dechlorinated water, resulted in complete parasite elimination. However, acute toxicity assay indicated a relatively narrow safety margin for prolonged bath treatments, with a 96 h LC_50_ of 0.039 mg/L, highlighting the need for caution when employing extended bath treatments. Oral administration of fenbendazole at 20 mg/kg body weight for three consecutive days resulted in an efficacy of 83.35%, which increased to 96.28% by seven days post-treatment. Safety evaluations revealed this regimen induced transient oxidative stress and mild, reversible histopathological alterations in the liver and gills. Biochemical and histological markers indicated a recovery trend, approaching baseline levels by 15 days post-treatment. These findings suggested that oral fenbendazole is an effective and relatively safe anthelmintic treatment against *G. kobayashii* in goldfish. This study underscores the potential of drug repurposing as an effective strategy for developing novel anthelmintic agents in aquaculture.

## Simple Summary

Parasitic infections are a major problem in fish farming, often causing illness and financial losses. In particular, tiny worms from the genus Monogenean can harm fish by attaching to their skin and gills. Currently, fish farmers have very few safe and effective medicines to treat these parasites, and repeated use of existing drugs may cause resistance. This study explored whether a common veterinary medicine called fenbendazole, usually used in terrestrial animals, could be an effective treatment for infected fish. The results showed that fenbendazole was highly effective at killing parasites, especially when given as a short bath or added to feed. Oral treatment for just three days removed over 96% of parasites, and the fish recovered well with only mild and temporary effects on their health. These findings suggest that fenbendazole could be a promising, practical, and safe option for treating fish parasites. Using this already-approved drug for a new purpose could offer a quicker, cheaper way to improve fish health and support sustainable aquaculture.

## 1. Introduction

The members of the genus *Gyrodactylus* Nordmann, 1832, a prevalent group of monogenean parasites, represent a major risk to both freshwater aquaculture and the health of wild fish populations [1,2]. These ectoparasites attach to the skin, fins, and gills of fish, causing mechanical damage, increased mucus production, fin erosion, and potentially leading to secondary infections and mortality, thereby resulting in substantial economic losses on fish farming [3,4]. The effective management of *Gyrodactylus* infections is therefore crucial for the sustainability and profitability of aquaculture. Currently, the control of monogenean diseases in aquaculture mainly relies on a limited number of legally approved therapeutic agents, primarily organophosphates such as trichlorfon and phoxim, and benzimidazoles like mebendazole [5,6]. These drugs have been used for a long time in the treatment of parasite infection and were officially upgraded from local to national standards around 2008, ensuring their legal availability across China [7]. However, since then, there has been an obvious absence of newly approved antiparasitic drugs for aquaculture in China. The prolonged lack of newly authorized treatments has raised growing concerns about the potential development of drug resistance in parasite populations, particularly among monogenean parasites, which are known to rapidly develop resistance due to their short life cycles, high reproductive rates, and frequent exposure to the same treatments [8,9]. Although this issue is prominent in China’s aquaculture, it reflects a broader global challenge. In many countries, limited drug options and overuse of existing treatments have similarly driven resistance, underscoring the need for safer, more effective alternatives [10,11].

Fenbendazole, a well-established broad-spectrum benzimidazole anthelmintic widely used in terrestrial veterinary medicine, has proven effective against a wide range of parasitic nematodes, cestodes, and trematodes [12,13]. This drug has been proven to target β-tubulin and then disrupts microtubule polymerization, inhibits the parasite’s glucose uptake and energy metabolism, ultimately leading to the death of the parasite [14,15]. Additionally, fenbendazole presents a promising candidate for application in aquaculture, exhibiting potent anthelmintic activity against monogeneans in fish. For instance, a therapeutic bath of fenbendazole (25 mg/L for two 12 h treatments with a 24 h break) effectively reduced *Dactylogyrus anchoratus* (Dujardin, 1845) infection (more than 90%) in juvenile common carp (*Cyprinus carpio*, Linnaeus, 1758) [11]. Similarly, oral administration of multiple doses of fenbendazole (20 mg/kg body weight on days 0, 3, and 7) demonstrated nearly 100% efficacy against gill parasites (*Dactylogyrus* sp.) from *Labeo rohita* (Hamilton, 1822) on the 15th day post-treatment [16]. Despite these promising findings, fenbendazole remains an unregistered drug for aquatic animals in China [6]. Its widespread use in human and terrestrial veterinary medicine, combined with the relatively low cost of raw materials and its demonstrated antiparasitic activity against aquatic parasites, makes it an ideal candidate for development as a legally approved anthelmintic drug in aquaculture [17]. However, before registering a new veterinary drug, a comprehensive preclinical trial is necessary, including determining the route of administration, and optimal dose of the drug, and assessing its potential impact on the host, which is crucial for ensuring the drug’s efficacy and safety.

Goldfish (*Carassius auratus*, Linnaeus, 1758) is a widely used model organism in parasitological and ecotoxicological research due to its hardiness and ease of maintenance [18]. Moreover, it serves as a natural host for *G. kobayashii* (Hukuda, 1940) and a well-established *G. kobayashii* infection model in goldfish has been developed in our laboratory [19]. This model enables consistent and reproducible experimental infections, providing a solid foundation for evaluating the therapeutic efficacy of fenbendazole under controlled conditions.

This study aimed to evaluate the anthelmintic efficacy and safety of fenbendazole against *G. kobayashii* in goldfish. In vivo assays were conducted to assess its anthelmintic activity and acute toxicity via bath administration. Additionally, oral administration was evaluated to determine the optimal dosage and treatment duration. The effects of the optimized treatment on liver and gill enzyme activities and histopathology were also investigated. These findings would provide essential preclinical data for the potential use of fenbendazole in the control of *Gyrodactylus* infections in aquaculture.

## 2. Materials and Methods

### 2.1. Fish, Parasites and Agents

Adult goldfish (mean weight: 3.41 ± 0.62 g; mean body length: 3.92 ± 0.73 cm) were procured from a local fish farm in Wuhan, China. Upon arrival at the laboratory, the fish were subjected to a two-week acclimation under controlled conditions. To eliminate any potential pre-existing ectoparasite infections, the goldfish underwent a series of formalin baths. Subsequently, a one-month recovery period was provided, during which a subset of goldfish was selected and examined for the presence of ectoparasites every 3 to 5 days to confirm their parasite-free status. Experimental fish were then obtained by co-culturing these parasite-free goldfish with individuals previously infected with *G. kobayashii*. The detailed experimental procedures followed in the present study have been documented in our prior publication [19,20]. Water quality parameters during fish husbandry were consistently maintained at the following levels: temperature, 22.3 ± 0.7 °C; pH, 7.15 ± 0.42; and dissolved oxygen, 6.1 ± 0.5 mg/L. Fenbendazole (purity ≥ 98%) and dimethyl sulfoxide (DMSO) were obtained from Shanghai Yuanye Biochemical Technology Co., Ltd., Shanghai, China.

### 2.2. In Vivo Anthelmintic Assay

Goldfish infected with *G. kobayashii* and exhibiting typical swimming behavior were selected. Parasite loads in these goldfish ranged from 40 to 200 worms per individual. These goldfish were randomly assigned to 1.5 L containers, each holding one fish in 0.5 L of dechlorinated water. Fenbendazole stock solution was prepared and diluted to achieve final concentrations of 0.004, 0.007, 0.01, 0.015, and 0.02 mg/L in the containers. In addition, two control groups were included: a negative control (drug-free) and a vehicle control (0.02% DMSO), both maintained under identical environmental conditions as those during the acclimation period. Each treatment and control group consisted of ten replicate goldfish, with each housed in a separate experimental container to minimize cross-contamination and allow for accurate observation of individual responses. Parasite enumeration was performed on the caudal fins of each fish before drug administration and again at 24 and 48 h post-treatment. Anthelmintic efficacy was determined using the established protocol outlined in our earlier publication [20].

To determine whether increased fenbendazole concentrations could reduce the required exposure time while maintaining comparable anthelmintic efficacy, a separate experiment was also conducted. In this trial, infected goldfish were exposed to higher fenbendazole concentrations ranging from 0.02 to 0.1 mg/L. Parasite loads were monitored hourly for a total duration of 6 h. These fish were then moved to dechlorinated water without the drug, and parasite loads were counted following an additional 18 h recovery interval. The experimental operations and the calculation of anthelmintic efficacy were conducted using the same methodology as outlined in the initial assay.

### 2.3. Acute Toxicity

To assess the potential toxicity of fenbendazole to goldfish, a 96 h static renewal bioassay was conducted [19]. Ten healthy, ectoparasite-free goldfish were randomly assigned to each of three replicate tanks, each containing 10 L of dechlorinated water. For the treatment groups, fenbendazole was added to achieve target concentrations of 0.02, 0.03, 0.04, 0.05, 0.06, and 0.08 mg/L. Two control groups were included: a negative control (drug-free) and a vehicle control (0.02% DMSO), both maintained under identical environmental conditions as those during the acclimation period. During the 96 h exposure, the mortality of goldfish was monitored. Any dead goldfish were immediately removed from the tanks, and the time of death was recorded. At the end of the experiment, the lethal concentration (LC_50_) of fenbendazole for goldfish was determined.

### 2.4. Oral Treatment with Fenbendazole

This study was conducted in two phases to determine the optimal dosage and treatment duration of orally administered fenbendazole for controlling *Gyrodactylus* infections in goldfish.

#### 2.4.1. Phase 1: Dose Screening (5-Day Treatment)

To assess the appropriate dosage of fenbendazole, a 5-day feeding trial was conducted. Commercial feeds were obtained from Jiangmen Qicai Pet Products Co., Ltd. (Guangzhou, China), and different doses of drugs were added to the feed to achieve the desired concentration (5, 10, 20, 40, 60, and 80 mg/kg body weight). The experiment was carried out in 500 L plastic tanks, each filled with 350 L of dechlorinated water, to minimize the potential impact of drug dispersal on anthelmintic efficacy. Five infected goldfish were randomly assigned to each tank. Before the start of the experiment, the total weight of the fish in each tank was recorded. Medicated feed was administered daily at a rate of 1% of total body weight for five consecutive days. Two control groups were included: a drug-free control, receiving drug-free feed; a vehicle control, receiving feed containing DMSO at the concentration used to dissolve fenbendazole. Each treatment group was replicated in three independent tanks. The parasite loads on the fins of each goldfish were evaluated and documented both before the start of treatment and at the end of the 5-day feeding period. During the experiment, water quality parameters were maintained at the levels established during the acclimation period.

#### 2.4.2. Phase 2: Time Course Evaluation (Optimal Dose)

Based on the results of Phase 1, the optimal fenbendazole dosage (10 and 20 mg/kg body weight) was selected for a time-course evaluation. The experimental setup, including tank size, water volume, number of fish per tank (five infected goldfish per tank), feeding rate (1% of body weight per day), and control groups (drug-free and DMSO-treated), remained consistent with the previous phase. Goldfish in the treatment group were fed daily with feed containing the optimal fenbendazole concentration. Parasite burdens on the fins were assessed and recorded at 3, 5, and 7 days post-treatment. The optimal dose and feeding time were determined by evaluating the anthelmintic efficacy.

### 2.5. Safety Evaluation of Oral Fenbendazole

The safety of oral fenbendazole to goldfish was evaluated over a 15-day feeding period. Healthy, ectoparasite-free goldfish (mean weight of 5.72 ± 0.84 g) were used for this experiment. The experiment was conducted in 500 L glass tanks, each containing 350 L of water. Before the start of the experiment, the total weight of the goldfish in each tank was recorded. Fish were fed daily with commercial feed at a rate of 1% of total body weight. The control group received standard feed without fenbendazole for 15 days, while the treatment group was given fenbendazole-medicated feed at 20 mg/kg body weight daily for the first 3 days, followed by standard feed for the remaining 12 days. Three replicate tanks (20 goldfish per tank) were used. Water quality parameters were maintained at the same levels as during the acclimation period during the experiment.

Tissue samples were collected at predetermined time points: before treatment and at 3, 6, 9, 12, and 15 days post-treatment. At each point, five goldfish were randomly selected, anesthetized with an overdose of MS-222, and their liver and gill tissues were immediately dissected. A portion of the liver and gill tissues was immediately frozen in liquid nitrogen and stored at −20 °C for subsequent analysis of enzyme activities. The remaining portions of the liver and gill tissues were fixed in a 4% formaldehyde solution for histopathological analysis.

For biochemical analysis, the frozen liver and gill tissues were homogenized in a cold physiological saline solution, followed by centrifugation (4000 g for 20 min at 4 °C), and the supernatants were collected for subsequent analysis. The activities of superoxide dismutase (SOD), catalase (CAT), glutathione S-transferase (GST), the level of malondialdehyde (MDA) and total protein content were determined using commercial kits from Nanjing Jiancheng Bioengineering Institute (Nanjing, China). Protein content was determined using the Bradford method, and all enzyme activities and MDA concentrations were normalized to total protein content [21]. Measurements were carried out using a spectrophotometer at the following wavelengths: 450 nm for SOD, 405 nm for CAT, 412 nm for GST, and 532 nm for MDA, according to the manufacturer’s protocols.

For histopathological analysis, the tissues were processed using standard histological procedures, including dehydration in a graded series of ethanol, clearing in xylene, and embedding in paraffin wax. Sections of 5 μm thickness were cut using a microtome, mounted on glass slides, and stained with hematoxylin and eosin (H&E) for microscopic examination. Histopathological changes in the liver and gill tissues were then evaluated under a light microscope (Eclipse Ci-L, Nikon, Tokyo, Japan).

### 2.6. Statistical Analysis

Statistical analyses were performed using R statistical software (version 4.3.3; R Core Team, 2024, Vienna, Austria). A significance level of *p* < 0.05 was used for all statistical tests. Probit analysis was used to calculate EC_50_ (effective concentration for 50% effect) and LC_50_ (lethal concentration for 50% mortality) based on the number of live and dead parasites or fish observed at each concentration of fenbendazole. Although efficacy was expressed as a percentage reduction in parasite numbers for presentation purposes, the underlying data used for Probit analysis were binary (alive vs. dead), making this approach appropriate for modeling the dose–response relationship. One-way ANOVA followed by Duncan’s multiple range tests was used to compare significant differences in the biochemical indexes among different treatment groups.

## 3. Results

### 3.1. In Vivo Anthelmintic Activity

**Figure 1:**
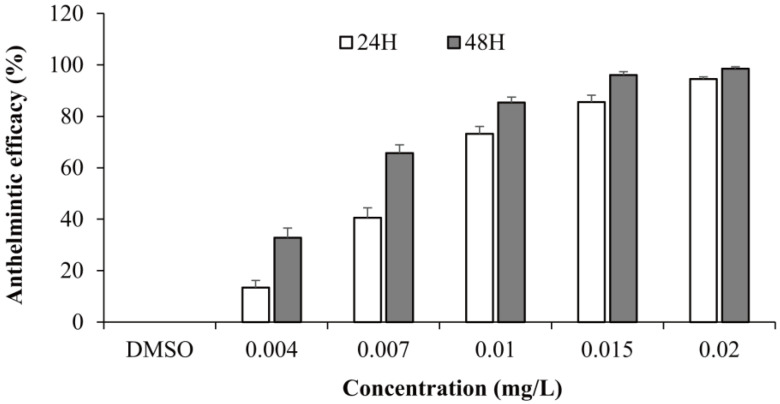
In vivo anthelmintic efficacy of fenbendazole against *Gyrodactylus kobayashii* in goldfish (*Carassius auratus*) after 24 and 48 h of exposure.

Anthelmintic efficacy of fenbendazole against *G. kobayashii* in goldfish was evaluated after 48 h of exposure. Fenbendazole exhibited dose-dependent anthelmintic activity against *G. kobayashii*. Anthelmintic efficacy ranged from 13.42% at 0.004 mg/L to 94.51% at 0.02 mg/L after 24 h of exposure. Extending the exposure to 48 h resulted in increased efficacy, ranging from 32.86% to 98.58% across the same concentration gradient (Figure 1). The calculated EC_50_ values were 0.009 mg/L (95% CI: 0.003–0.012 mg/L) and 0.006 mg/L (95% CI: 0.004–0.006 mg/L) after 24 and 48 h of exposure, respectively. In the control group, no reduction in parasite loads was observed.

**Figure 2:**
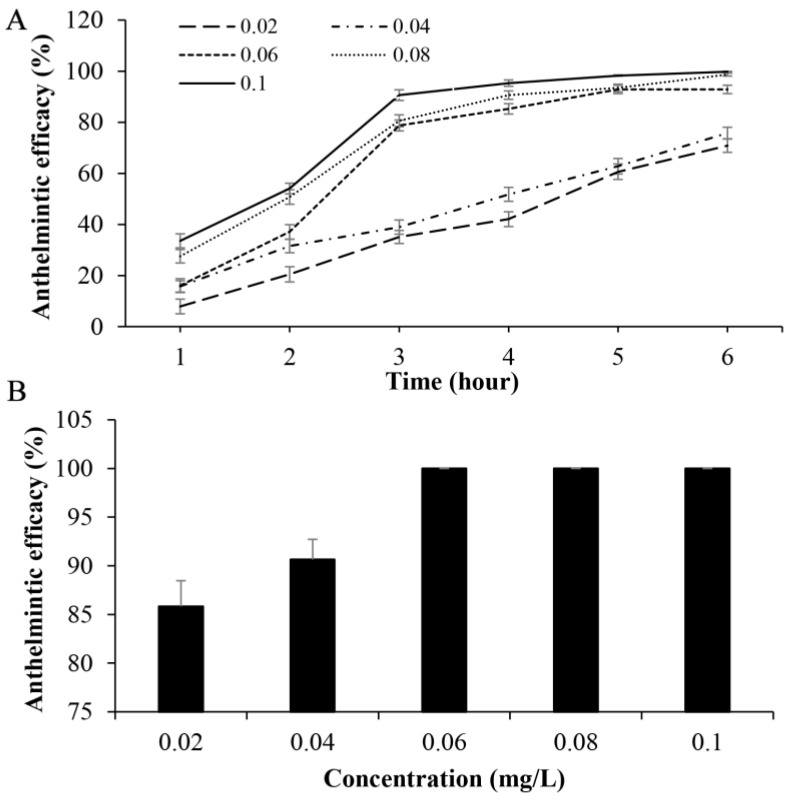
(**A**) In vivo anthelmintic efficacy of fenbendazole against *Gyrodactylus kobayashii* in goldfish (*Carassius auratus*) after 6 h exposure. (**B**) In vivo anthelmintic efficacy of 6 h exposure to different concentrations of fenbendazole followed by an 18 h recovery period in dechlorinated water.

To further explore the potential for shorter treatment durations with higher drug concentrations, infected goldfish underwent a 6 h bath in elevated fenbendazole concentrations, followed by an 18 h recovery period in dechlorinated water. Anthelmintic efficacy of fenbendazole during the initial 6 h bath is detailed in Figure 2. At the lowest concentration of 0.02 mg/L, anthelmintic efficacy gradually increased to 70.84% by the end of the 6 h exposure. Similarly, anthelmintic efficacy of 75.77% was observed at 0.04 mg/L after 6 h exposure. Higher concentrations demonstrated more rapid and potent activity within the 6 h exposure. Anthelmintic efficacy achieved 78.73% at 0.06 mg/L after 3 h of exposure, reaching a plateau of 92.85% after 5 h of exposure. Higher anthelmintic efficacies of 98.66 and 99.78% were achieved at concentrations of 0.08 and 0.1 mg/L after 6 h exposure, respectively (Figure 2A). After the subsequent 18 h recovery period in dechlorinated water, complete parasite elimination (100% efficacy) was observed at fenbendazole concentrations of 0.06 mg/L and above (Figure 2B).

### 3.2. Acute Toxicity

As shown in Table 1, mortality was generally low across the tested concentrations within the initial 48 h of exposure. During the first 24 h exposure, mortality was observed only at concentration of 0.08 mg/L, with an average of 1.33 dead fish per tank. By the end of the 48 h exposure, mortality remained relatively low, with a maximum of 3.33 dead fish per tank at a concentration of 0.06 mg/L. However, a notable increase in mortality occurred during subsequent exposure. Substantial mortality was observed after 72 h of exposure, ranging from an average of 6.0 dead fish per tank at 0.04 mg/L to 10.0 dead fish per tank at 0.08 mg/L. By the end of the 96 h exposure, mortality reached 100% at concentrations of 0.06 mg/L and 0.08 mg/L. In contrast, no mortality occurred in the control groups during the entire 96 h exposure. The 48 and 96 h LC_50_ of fenbendazole for goldfish was calculated to be 0.073 mg/L (95% CI: 0.063–0.098 mg/L) and 0.039 mg/L (95% CI: 0.034–0.044 mg/L), respectively.

### 3.3. Oral Administration with Fenbendazole

The efficacy of orally administered fenbendazole against *G. kobayashii* in goldfish was evaluated. In the 5-day dose-screening trial (Table 2), fenbendazole demonstrated a dose-dependent reduction in the infection abundance of *G. kobayashii* and a corresponding increase in anthelmintic efficacy after a 5-day treatment. Anthelmintic efficacy ranged from 38.24% at 5 mg/kg body weight to over 90% at 20 mg/kg and above. In contrast, the drug-free and vehicle control groups exhibited high infection abundance at the end of the 5-day treatment (100.73 ± 12.11 and 97.60 ± 7.85, respectively).

Based on the dose-screening results, oral administrations of fenbendazole at 10 and 20 mg/kg body weight were selected for the time-course evaluation (Table 3). Anthelmintic efficacy demonstrated a dose- and time-dependent manner. At a dose of 10 mg/kg body weight, anthelmintic efficacy increased from 36.21% at 3 days post-treatment to 74.31% by 7 days post-treatment. A higher dose of 20 mg/kg body weight resulted in higher efficacy at all time points, reaching 83.35% at 3 days post-treatment, 93.83% at 5 days post-treatment, and 96.28% at 7 days post-treatment. In the control groups, infection abundance of *G. kobayashii* in goldfish increased. Considering both efficacy and cost-effectiveness, oral administration of fenbendazole at 20 mg/kg body weight for three days is recommended.

### 3.4. Safety Evaluation of Oral Administration with Fenbendazole

#### 3.4.1. Biochemical Analysis

**Figure 3:**
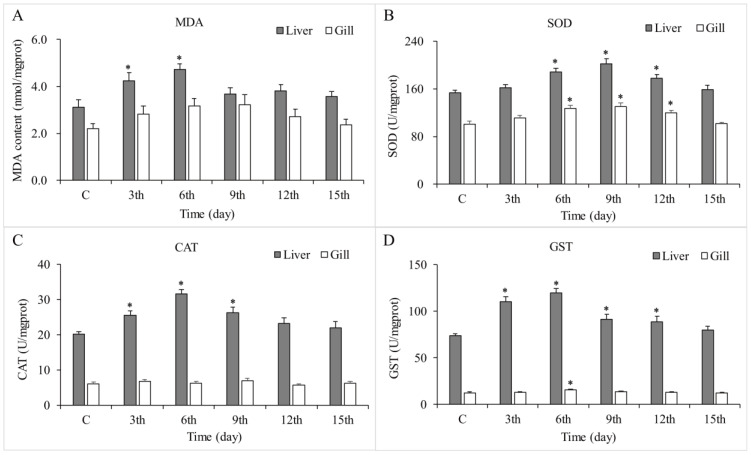
The biochemical markers of oxidative stress, (**A**) malondialdehyde, (**B**) superoxide dismutase, (**C**) catalase, and (**D**) glutathione S-transferase, in liver and gill tissues of goldfish (*Carassius auratus*) after an oral dose of 20 mg/kg of fenbendazole for 3 consecutive days over a 15-day course. Data are presented as means ± SE, with n = 5. Asterisk (*) indicates a significant difference between the treatment group and the control group (*p* < 0.05).

The safety of oral fenbendazole (20 mg/kg for 3 consecutive days) to goldfish was evaluated by assessing biochemical markers of oxidative stress in liver and gill tissues over a 15-day course (Figure 3). MDA levels demonstrated an initial increase in the liver tissue following fenbendazole administration, peaking significantly at 3 days post-treatment compared to the control group (*p* < 0.05). Subsequently, MDA levels gradually decreased and returned closer to control levels by 15 days post-treatment. SOD activity in the liver also exhibited an increasing trend after fenbendazole administration, peaking around 6 days post-treatment, significantly higher than the control group (*p* < 0.05). SOD activity remained elevated through 9 days post-treatment and then gradually decreased and appeared to return to control levels by 15 days post-treatment. Likewise, CAT and GST activities exhibited a similar trend: both enzymes initially increased, reaching peak activity within the first 6 days post-treatment, and then decreased, returning to baseline levels by the end of the 15-day treatment.

In the case of gill tissues, MDA levels showed fluctuations following fenbendazole administration. There was an initial increase at 3 days post-treatment and then returned to control levels or slightly below at 6 days post-treatment. The activities of antioxidant enzymes (SOD, CAT, GST) showed a notable increase after treatment, peaking around 6 to 9 days post-treatment, and then declined and approached baseline levels by the end of the 15-day treatment.

#### 3.4.2. Histopathological Analysis

**Figure 4:**
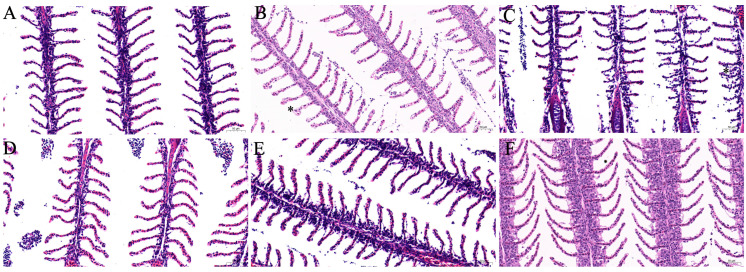
Histopathological analysis of the gill of goldfish (*Carassius auratus*) following oral administration of fenbendazole at 20 mg/kg body weight for three consecutive days over a 15-day course. (**A**) Control group gills from goldfish fed drug-free feed. (**B**–**F**) Gills from goldfish treated with 20 mg/kg fenbendazole for 3 days, sampled at (**B**) 3 days post-treatment, (**C**) 6 days post-treatment, (**D**) 9 days post-treatment, (**E**) 12 days post-treatment, and (**F**) 15 days post-treatment. Asterisk indicates hyperplasia.

The histopathological characteristics of the gills after oral administration with fenbendazole are presented in Figure 4. The gills of the control goldfish displayed a normal structure (Figure 4A). In contrast, the treated goldfish maintained the overall structural integrity of their gills, with only limited hyperplasia observed at 3 and 6 days post-treatment.

**Figure 5:**
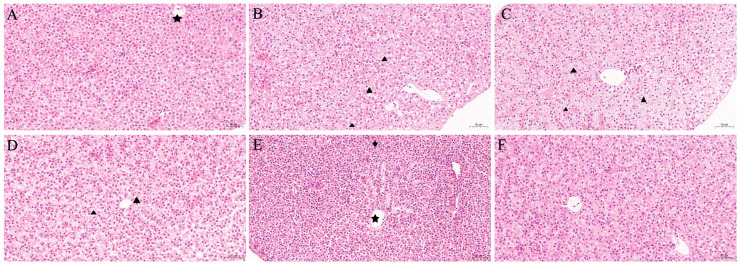
Histopathological analysis of the liver of goldfish (*Carassius auratus*) following oral administration of fenbendazole at 20 mg/kg body weight for three consecutive days over a 15-day course. (**A**) Control group gills from goldfish fed drug-free feed. (**B**–**F**) Gills from goldfish treated with 20 mg/kg fenbendazole for 3 days, sampled at (**B**) 3 days post-treatment, (**C**) 6 days post-treatment, (**D**) 9 days post-treatment, (**E**) 12 days post-treatment, and (**F**) 15 days post-treatment. The star indicates the central vein, the triangle indicates cytoplasmic vacuolization, and the diamond indicates pigment deposition.

As shown in Figure 5, the hepatic architecture of the control group (Figure 5A) appeared normal, with tightly arranged hepatocytes displaying consistent morphology and no evidence of tissue injury or inflammation. In comparison, goldfish that received oral fenbendazole exhibited evident cytoplasmic vacuolization and pigment deposition (Figure 5B–F). However, the extent of vacuolization gradually decreased over time, suggesting a trend toward recovery.

## 4. Discussion

The disease caused by parasites represents a significant challenge to aquaculture, frequently resulting in substantial economic losses [22,23]. Current control strategies mainly rely on a limited number of approved anthelmintic agents, many of which have been used for several decades [5,24]. The prolonged and frequent use of these agents exerts considerable selection pressure on parasite populations, inevitably leading to the emergence of drug resistance and reducing the effectiveness of existing treatment strategies [8,9]. Therefore, there is a pressing need for the development of novel or alternative therapeutic agents. However, the development and regulatory approval of novel therapeutic agents for aquatic parasites have faced considerable challenges. The registration of new veterinary drugs is a complex and resource-intensive process, which requires the completion of numerous experiments, including pharmacodynamics, pharmacokinetics, toxicology, and clinical trials, to verify both their effectiveness and safety [25]. The extensive evaluations require significant investment in both manpower and material resources. However, considering the relatively small market size of the novel therapeutic agents for aquatic parasites compared to terrestrial animal or human pharmaceuticals, as well as the huge investment, many companies are not particularly willing to register new veterinary agents for aquatic parasites [26].

Given the significant obstacles associated with registering new veterinary agents for aquatic parasites, the strategy of drug repurposing provides a practical and effective alternative [27,28]. This approach mainly evaluates existing drugs approved for use in humans or terrestrial animals for new applications against parasites of aquatic animals [29]. The main advantage lies in using the extensive information already prepared for the original approval, including pharmacodynamics, toxicology, and mature production processes. Compared to developing entirely new drugs, drug repurposing significantly shortens drug development time and lowers overall costs and associated risks [30,31]. Therefore, for the veterinary agents for aquatic parasites with a limited market, drug repurposing is a very feasible and effective strategy that can accelerate the availability of urgently needed and effective treatment options. Therefore, this study explored the feasibility of this strategy by evaluating the potential of fenbendazole, a widely used benzimidazole anthelmintic, in controlling *G. kobayashii* infections in goldfish.

In this trial, in vivo assays demonstrated that fenbendazole exhibited potent anthelmintic activity against *G. kobayashii* in a dose-dependent manner. Anthelmintic efficacy reached 98.58% at concentration of 0.02 mg/L, with an EC_50_ value of 0.006 mg/L after 48 h of exposure. The anthelmintic efficacy is significantly higher than that of several other agents previously reported for the treatment of gyrodactylids, such as isoimperatorin (EC_50_ = 0.53 mg/L), plumbagin (EC_50_ = 0.09 mg/L), and the commonly used anthelmintic mebendazole (EC_50_ = 0.023 mg/L) [32,33,34]. These findings highlighted the strong potential of fenbendazole as an effective anthelmintic agent.

Directly dispersing drugs into the water is the most common method for treating fish ectoparasites in pond farming, the dominant form of aquaculture in China [35]. Despite its simplicity, the method has considerable drawbacks, including low drug utilization efficiency, environmental pollution, and increased risk of drug resistance. With the rise in large-scale, standardized systems, particularly controlled recirculating aquaculture, high-concentration bath treatments for short duration have emerged as a preferred alternative [36]. This method offers precise administration and rapid parasite elimination with minimal potential adverse effects on fish, prevents environmental discharge, and allows recycling to reduce overall costs. In this trial, baths with 0.06 mg/L fenbendazole for 6 h, followed by transfer to dechlorinated water, led to the complete elimination of *Gyrodactylus* in goldfish within 18 h post-treatment. A similar “post-treatment effect” has also been reported for febantel, a prodrug of fenbendazole, where short-term (6 h) exposure followed by transfer to drug-free water still led to complete parasite elimination [37].

The delayed anthelmintic effect observed may be explained by the mode of action of benzimidazoles, which impair energy metabolism in parasites, resulting in progressive energy depletion and eventual death [38,39]. Since energy exhaustion in parasites is a cumulative, time-dependent process, this mechanism may account for the sustained anthelmintic efficacy or “post-treatment effect” observed even after the drug has been removed. These findings further support the feasibility of short-duration exposure strategies, as demonstrated in our study and previous reports on febantel, where parasite elimination continued during the post-treatment period in drug-free water [37]. However, contrasting results were reported by Tojo et al. [40], who found that a 12 h in vivo exposure to 1.5 mg/L fenbendazole was required to eliminate *G. salaris* (Malmberg, 1957), while an even higher concentration (12.5 mg/L) was ineffective after a 1 h in vitro exposure. In this trial, a much lower concentration (0.06 mg/L) for a 6 h in vivo exposure, followed by transfer to dechlorinated water, resulted in complete elimination of *G. kobayashii* in goldfish within 18 h post-treatment. These discrepancies may be attributed to the species-specific differences in parasite susceptibility (*G. kobayashii* vs. *G. salaris*) and variations in experimental conditions. The purity and solvent of fenbendazole were not provided in the previous study by Tojo et al. [40], whereas fenbendazole with ≥98% purity dissolved in DMSO was used in this trial. The differences in drug formulation can significantly influence bioavailability and, consequently, therapeutic efficacy.

The safety profile of the drugs is an important factor in determining their application in aquaculture [41]. In this trial, acute toxicity of fenbendazole administered via baths to goldfish was assessed. The results showed that mortality remained relatively low during the initial 48 h of exposure, even at the high concentration of 0.06 mg/L. However, a marked increase in mortality was observed thereafter, reaching 100% after 96 h at the same concentration. This delayed toxicity profile is consistent with the established mechanism of action of benzimidazole anthelmintics, involving the gradual disruption of energy metabolism in target organisms. Additionally, acute toxicity assays indicated that the 48 h LC_50_ was 0.073 mg/L, resulting in a therapeutic index (TI, LC_50_/EC_50_) of approximately 12.17. However, when considering the concentration required for nearly 100% anthelmintic efficacy (98.59 efficacy at concentration of 0.02 mg/L after 48 h exposure), the safety margin narrows to about 3.65. These findings suggested that despite the demonstrated anthelmintic efficacy of fenbendazole administered via baths, its limited safety margin under prolonged exposure conditions (≥48 h) raises concerns about its suitability for extended use for goldfish. In contrast, short-term bath treatments (e.g., 6 h) at high concentrations (e.g., 0.06 mg/L), which have demonstrated complete efficacy against *G. kobayashii*, may offer a more favorable safety profile. However, further studies are necessary to validate the safety profile of a short-term treatment regimen.

In comparison with bath treatment, oral administration is a promising option in aquaculture due to its operational simplicity, reduced stress on fish, and lower environmental impact [42]. In this study, the efficacy of orally administered fenbendazole against *G. kobayashii* in goldfish was evaluated. The results demonstrated that fenbendazole displayed a clear dose- and time-dependent anthelmintic effect, and oral administration of fenbendazole at 20 mg/kg body weight for 3 days resulted in an anthelmintic efficacy of 83.35%, which increased to 96.28% following a 7-day treatment. While extended treatment periods may enhance anthelmintic efficacy, shorter regimens with reduced dosages offer advantages such as lower costs, decreased withdrawal periods, and minimized risks to fish health and aquatic ecosystems. Moreover, complete parasite elimination may not always be necessary. When parasite loads are sufficiently reduced, fish can often rely on their immune systems to manage residual infections, thereby maintaining health without intensive drug intervention [43]. Furthermore, complete parasite elimination is not always necessary. When the parasite burden is sufficiently reduced, fish can often rely on their immune system to eliminate the remaining parasites. Thus, considering both therapeutic efficacy and practical applicability, oral administration of fenbendazole at 20 mg/kg body weight for three days appears to provide an optimal balance between effectiveness and cost-efficiency.

To evaluate the safety of the optimized oral dosage and treatment duration, goldfish were administered fenbendazole orally at 20 mg/kg body weight for three consecutive days. Subsequently, biochemical markers and histopathological changes in the gills and liver were assessed. Biochemical analysis revealed that fenbendazole induced a transient oxidative stress response in both tissues, particularly in the liver. This was evidenced by elevated activities of antioxidant enzymes (SOD, CAT, and GST), which suggested activation of the antioxidant defense system in response to increased reactive oxygen species [44]. Additionally, elevated MDA levels, especially in the liver, suggested lipid peroxidation and some degree of oxidative damage. However, by the end of the observation period, most biochemical parameters appeared to be returning toward baseline levels, suggesting a trend of recovery. Histopathological analysis revealed tissue-specific responses consistent with the biochemical findings. The gills exhibited only mild hyperplasia at early time points and largely retained their structural integrity. In contrast, the liver showed more obvious alterations, characterized by cytoplasmic vacuolization. These hepatic changes may be attributed to its role as a primary metabolic organ, potentially making it a target for fenbendazole or its metabolites. Importantly, the vacuolization decreased over the 15-day period, consistent with the recovery observed in biochemical markers. Overall, oral administration of fenbendazole at 20 mg/kg body weight for three days induced temporary oxidative stress and mild histological changes in goldfish, particularly in the liver. Both biochemical and histopathological findings suggest these effects are reversible, supporting the safety of the tested treatment regimen.

## 5. Conclusions

In summary, this study confirmed that fenbendazole, an anthelmintic widely used in terrestrial animals, exhibits potent anthelmintic efficacy against *G. kobayashii* in goldfish through both bath and oral administration. Bath treatment is highly effective, especially the short-term, high-concentration bath combined with its post-treatment effect, offering a promising therapeutic option potentially suitable for controlled recirculating aquaculture systems. However, the narrow safety margin associated with prolonged baths may limit its broader application. Oral administration offers a more favorable safety profile and operational convenience, providing a viable alternative for large-scale farms or situations where bathing is not feasible. Importantly, these findings support the strategy of drug repurposing—re-evaluating existing drugs for potential applications in aquaculture—as a practical approach to address the limited number of approved antiparasitic agents in aquaculture.

## Figures and Tables

**Table 1:** Acute toxicity of fenbendazole against goldfish (*Carassius auratus*), (Mean ± SE, N = 3).

Concentration (mg/L)	No. of Fish/Tank	No. Dead Fish (Mean ± SE)			
		24 h	48 h	72 h	96 h
0	10	0	0	0	0
0.02% DMSO	10	0	0	0	0
0.02	10	0	0	0	0
0.03	10	0	0	0	0.67 ± 0.33
0.04	10	0	1.33 ± 0.33	6.0 ± 0.58	7.33 ± 0.33
0.05	10	0	1.67 ± 0.33	7.67 ± 0.67	8.0 ± 0.58
0.06	10	0	3.33 ± 0.33	8.33 ± 0.33	10
0.08	10	1.33 ± 0.33	5.67 ± 0.33	10	10

**Table 2:** Effect of oral administration with different concentrations of fenbendazole on *Gyrodactylus kobayashii* infection in goldfish (*Carassius auratus*). Five goldfish per replicate and three replicates were used for each treatment.

Treatment (mg/kg Body Weight)	Infection Abundance (Mean ± SE)		Anthelmintic Efficacy (%, Mean ± SE)
	0th	5th	
drug-free control group	58.87 ± 6.69	100.73 ± 12.11	/
vehicle control group	53.40 ± 4.54	97.60 ± 7.85	/
5	66.67 ± 5.25	42.40 ± 5.28	38.24 ± 4.57
10	59.80 ± 8.21	21.40 ± 4.71	64.08 ± 3.58
20	62.27 ± 8.01	4.47 ± 0.98	91.76 ± 2.17
40	63.47 ± 5.44	1.93 ± 0.44	96.41 ± 0.98
60	60.60 ± 7.46	0.93 ± 0.54	98.55 ± 0.73
80	59.00 ± 8.39	0.67 ± 0.30	98.83 ± 0.56

**Table 3:** Effect of oral administration with optimal doses of fenbendazole at different time points on *Gyrodactylus kobayashii* infection in goldfish (*Carassius auratus*). Five goldfish per replicate and three replicates were used for each treatment.

Treatment (mg/kg Body Weight)	Infection Abundance (Mean ± SE)				Anthelmintic Efficacy (%, Mean ± SE)		
	0th	3th	5th	7th	3th	5th	7th
0	56.20 ± 4.96	74.07 ± 5.61	92.73 ± 6.91	122.13 ± 8.42	/	/	/
DMSO	60.07 ± 6.04	81.40 ± 7.25	97.67 ± 6.74	127.53 ± 11.79	/	/	/
10	62.47 ± 6.03	39.20 ± 4.14	19.47 ± 2.53	15.60 ± 2.44	36.21 ± 3.77	66.43 ± 4.09	74.31 ± 3.08
20	57.33 ± 6.17	8.93 ± 0.81	3.20 ± 0.54	1.87 ± 0.47	83.35 ± 1.61	93.83 ± 1.17	96.28 ± 0.91

## Data Availability

The datasets used and analyzed during the current study are available from the corresponding author upon reasonable request.
